# Ferulic Acid Modulates High Glucose-Induced MMP-1/TIMP-1 Imbalance and Enhances Wound Closure of Human Gingival Fibroblasts

**DOI:** 10.3390/biomedicines14081803

**Published:** 2026-08-11

**Authors:** Akın Özdemir, Ali Yüncü, Ayşe Mine Yılmaz, Hafize Öztürk Özener

**Affiliations:** 1 Department of Periodontology, Faculty of Dentistry, Marmara University, Maltepe, Istanbul 34854, Turkey; hafize.ozturk@marmara.edu.tr; 2 Department of Biochemistry, School of Medicine, Marmara University, Maltepe, Istanbul 34854, Turkey; ali.yuncu@marun.edu.tr (A.Y.); ayse.mine@marmara.edu.tr (A.M.Y.)

**Keywords:** fibroblasts, extracellular matrix, wound healing, ferulic acid, diabetes mellitus, hyperglycemia, tissue inhibitor of metalloproteinase-1

## Abstract

**Background/Objectives**: Chronic hyperglycemia drives gingival fibroblast dysfunction, disrupting extracellular matrix homeostasis and the migratory capacity required for wound healing, which contributes to increased periodontal susceptibility in diabetes. Ferulic acid (FA), a dietary phenolic compound with antioxidant and anti-inflammatory properties, has demonstrated wound-healing activity in various tissues; however, its effects on human gingival fibroblasts (HGFs) under hyperglycemic conditions remain unclear. This study investigated the effects of FA on HGF viability, wound closure, and the secreted concentrations of matrix metalloproteinase-1 (MMP-1), tissue inhibitor of metalloproteinase-1 (TIMP-1), and interleukin-6 (IL-6) under normoglycemic and hyperglycemic conditions. **Methods**: Cultured HGFs (HGF-1 cell line) were pre-conditioned for 72 h under normal glucose (5.5 mM) or high glucose (25 mM), with an iso-osmolar mannitol control to distinguish glucose-specific effects from osmotic changes. Viability was evaluated by 3-(4,5-dimethylthiazol-2-yl)-2,5-diphenyltetrazolium bromide (MTT) assay and wound closure by scratch assay following FA treatment, while MMP-1, TIMP-1, and IL-6 concentrations in conditioned medium were quantified by enzyme-linked immunosorbent assay (ELISA). **Results**: FA was non-cytotoxic at all tested concentrations, whereas 50 µM transiently increased viability. FA significantly enhanced wound closure under both conditions, independent of osmotic effects. High glucose increased IL-6 concentration in the conditioned medium (significant at 48 h) and MMP-1 levels. FA partially attenuated MMP-1 levels at specific time points, modestly reduced IL-6 levels, and markedly increased TIMP-1 concentration under both glucose conditions. **Conclusions**: FA enhanced wound closure in scratch assay and shifted the secreted MMP-1/TIMP-1 concentration ratio toward a more favorable, matrix-protective profile at the protein level, without cytotoxicity, supporting further investigation of its potential role in periodontal tissue repair under high glucose conditions.

## 1. Introduction

Diabetes mellitus is a metabolic disorder characterized by chronic hyperglycemia, which impairs the function of multiple cell types involved in tissue repair. Chronic hyperglycemia produces a broad spectrum of complications, including retinopathy, nephropathy, neuropathy, cardiovascular disease, and impaired wound healing, that share a common basis in microvascular injury, oxidative stress, and dysregulated tissue repair [1]. Among these, periodontal disease has long been recognized as the sixth complication of diabetes [2], and it is driven largely by gingival fibroblast dysfunction, which makes the gingival compartment a clinically important and mechanistically accessible target. Gingival wound healing is particularly vulnerable to hyperglycemic conditions, as human gingival fibroblasts (HGFs) are the predominant stromal cells responsible for extracellular matrix (ECM) remodeling, collagen synthesis and wound closure in periodontal tissues [3,4]. Epidemiological and experimental evidence demonstrates that diabetic patients exhibit delayed gingival wound healing, increased susceptibility to periodontal infections and impaired post-surgical recovery [4,5,6,7]. Recent evidence further confirms that the bidirectional relationship between diabetes and periodontal disease is sustained by a vicious cycle of chronic inflammation, immune dysfunction and microbial dysbiosis, in which poor glycemic control aggravates periodontal destruction while periodontal inflammation worsens glycemic control [8].

The molecular basis of hyperglycemia-induced HGF dysfunction has been investigated in model systems using glucose concentrations that reflect clinically observed postprandial levels. Exposure of HGFs to 25 mM glucose for 72 h significantly reduces cell proliferation and migration and is associated with increased expression of oxidative stress markers, including heme oxygenase-1 and superoxide dismutase-1 [9]. Consistent with these findings, diabetes reduces fibroblast numbers in gingival wounds through increased apoptosis and decreased proliferation, mechanisms that are mediated, at least in part, by FOXO1 activation [10]. Together, these findings support the use of 25 mM glucose as a biologically relevant model of diabetic hyperglycemia in HGF cultures, as it induces specific and reproducible cellular dysfunction without the excessive osmotic toxicity observed at higher concentrations [9].

A critical consequence of chronic hyperglycemia is the dysregulation of matrix metalloproteinases (MMPs) and their endogenous inhibitors, tissue inhibitors of metalloproteinases (TIMPs). Increased expression of MMP-1, MMP-2, MMP-8, and MMP-9 has been reported in diabetic wound tissues, contributing to excessive ECM degradation and impaired wound matrix formation [11,12]. This proteolytic imbalance is further exacerbated by the concurrent downregulation of TIMP-1 and TIMP-2 in diabetic wound fibroblasts, sustaining a catabolic microenvironment that impairs tissue repair [13,14]. In the periodontium specifically, MMP-1, MMP-2, MMP-9 and MMP-13 have been identified as key collagenolytic enzymes whose increased activity, coupled with insufficient TIMP-mediated inhibition, contributes to ECM degradation [15]. In addition, elevated IL-6 concentration by HGFs under high-glucose conditions sustains a pro-inflammatory microenvironment that further compromises gingival wound healing. Hyperglycemia and its downstream products, including advanced glycation end-products, have been shown to increase IL-6 production in HGFs, thereby prolonging the inflammatory phase and suppressing the cellular functions required for effective tissue repair [16,17]. Elevated IL-6 levels in the periodontal microenvironment have also been linked to enhanced MMP release and pathological ECM breakdown, reinforcing the central role of this cytokine in connecting hyperglycemia-driven inflammation to tissue destruction [18].

Ferulic acid (4-hydroxy-3-methoxycinnamic acid) (FA) is a naturally occurring phenolic acid belonging to the hydroxycinnamic acid family. It is biosynthesized via the phenylpropanoid pathway in plants and is widely distributed in cereal grains, fruits, and vegetables, where it is predominantly esterified to cell wall polysaccharides [19]. FA exerts well-characterized antioxidant activity through scavenging reactive oxygen species and inhibiting lipid peroxidation [20]. Beyond its antioxidant properties, FA has demonstrated anti-inflammatory, antimicrobial, and antidiabetic effects in various experimental models [21]. Given its low cytotoxicity and favorable biological profile, FA has attracted considerable interest as a therapeutic agent in dermatology, wound healing, and metabolic disease research [22,23]. Recent comprehensive reviews have further highlighted FA’s multifaceted role in modulating glucose and lipid metabolism, oxidative stress and inflammatory and microbiota-related pathways, supporting its potential application in metabolic syndrome and its complications [24].

Despite this converging evidence, these effects have been studied only in isolation: ferulic acid has not been evaluated as a single compound in human gingival fibroblasts under high-glucose conditions with an iso-osmolar mannitol control, and its influence on the MMP-1/TIMP-1 balance in this setting remains undefined. The present study therefore aimed to investigate the effects of FA on HGF viability, wound closure, and the secreted concentrations of MMP-1, TIMP-1, and IL-6 under normoglycemic and hyperglycemic conditions.

## 2. Materials and Methods

### 2.1. Cell Culture

The HGF-1 line of HGFs (ATCC, CRL-2014; Manassas, VA, USA) served as the experimental model. Cultures were propagated in Dulbecco’s Modified Eagle’s Medium (DMEM; Biochrom AG, Berlin, Germany) containing 10% fetal bovine serum (FBS; Gibco, Thermo Fisher Scientific, Waltham, MA, USA) and penicillin–streptomycin (Gibco, Thermo Fisher Scientific, Waltham, MA, USA) added at 1%, giving final concentrations of 100 U/mL penicillin and 100 µg/mL streptomycin. The DMEM formulation used contained stable L-glutamine; no additional L-glutamine was supplemented. Cells were maintained in a humidified 5% CO_2_ incubator (Sanyo MCO-18AIC; Sanyo Electric Co., Ltd., Osaka, Japan) at 37 °C, and all experiments were performed with cells at passages 4–5. A 100 mM stock of ferulic acid (Glentham Life Sciences Ltd., Corsham, UK; CAS: 1135-24-6) was first solubilized in dimethyl sulfoxide (DMSO; Sigma-Aldrich Co., St. Louis, MO, USA), and the required working dilutions were freshly prepared in medium before each use. Across all experiments, DMSO in the final medium was kept at or below 0.1%.

### 2.2. Establishment of the Hyperglycemic Cell Culture Model

Normoglycemic (NG; 5.5 mM D-glucose) and hyperglycemic (HG; 25 mM D-glucose) conditions were established by culturing cells in DMEM supplemented with the respective glucose concentrations. Three control conditions are used throughout: the vehicle control (0.1% DMSO, used to normalize viability), the untreated control (FA-free cells at the relevant glucose condition), and the iso-osmolar mannitol control (5.5 mM D-glucose + 19.5 mM D-mannitol; AppliChem GmbH, Darmstadt, Germany). Cells were pre-exposed to the assigned glucose conditions for 72 h prior to all functional assays.

### 2.3. Cell Viability Assay (MTT)

For viability testing, HGFs were plated in 96-well flat-bottom microtiter plates at 5 × 10^3^ cells per well and held in a humidified 5% CO_2_ incubator at 37 °C. Using normal-glucose DMEM, the cells then received FA across a concentration range of 5, 10, 25, 50, and 100 µM over 24, 48, and 72 h periods. Working solutions were obtained by dissolving FA in dimethyl sulfoxide (DMSO; Sigma-Aldrich Co., St. Louis, MO, USA) and diluting in medium, while a parallel vehicle control carrying 0.1% DMSO was run throughout to correct for any solvent contribution. When each interval elapsed, every well received 10 µL of MTT reagent (Thermo Fisher Scientific, Waltham, MA, USA; 5 mg/mL in phosphate-buffered saline; Wisent Inc., St-Bruno, QC, Canada), and the plates were returned to 37 °C for 4 h. The formazan that formed was subsequently solubilized with 100 µL DMSO per well, and absorbance was read at 570 nm on a PerkinElmer EnSpire 2300 Multilabel Plate Reader (PerkinElmer, Boston, MA, USA).

Viability was expressed relative to the 0.1% DMSO vehicle control, which was set to 100%, according to:

Cell viability (%) = [(OD_sample − OD_blank)/(OD_vehicle control − OD_blank)] × 100

### 2.4. Scratch Assay

In this study, wound closure is defined operationally as the reduction in the cell-free (denuded) area created by scratching a confluent monolayer, and was evaluated with an *in vitro* scratch assay based on the method of Liang et al. [25], following the procedure previously established in our laboratory with modifications to incorporate the glucose pre-conditioning step [26]. HGFs were seeded at a density of 1.5 × 10^5^ cells/well in 6-well flat-bottom plates and cultured in 2 mL of complete growth medium. Following 72 h of glucose pre-conditioning, the medium was removed and a uniform linear scratch was created in the center of each monolayer using a sterile 200 µL pipette tip. Wells were washed twice with PBS to remove detached cells and debris. Experimental medium consisting of complete DMEM (10% FBS) containing the assigned glucose concentration and FA at 25 and 50 µM was then applied. Because proliferation was not pharmacologically inhibited, wound closure in this assay reflects the combined contribution of cell migration and proliferation. These concentrations were selected on the basis of the MTT screen: although all tested concentrations (5–100 µM) were non-cytotoxic, 5–10 µM produced minimal change in metabolic activity, whereas 50 µM elicited the peak response at 24 h and 25 µM represented an intermediate active dose, while 100 µM produced no greater response than 50 µM and was therefore not carried forward. The 25 and 50 µM concentrations therefore bracket the biologically active, non-cytotoxic window while permitting evaluation of a concentration effect. Separate plates were assigned to each time point, both to avoid repeated removal of cultures from the incubator and to permit immediate harvesting of conditioned media for the ELISA analyses. Each plate was imaged with a phase-contrast inverted light microscope immediately after scratching (0 h) and again at its assigned endpoint (24, 48, or 72 h), at which point it was removed from the incubator and the conditioned medium harvested. Images from different endpoints therefore derive from different plates rather than from sequential imaging of a single wound field, and imaging was not performed at fixed stage coordinates. Residual wound area was measured manually in ImageJ (version 1.54j, National Institutes of Health, Bethesda, MD, USA) using the standard selection tools, without an automated wound-healing macro, and reported as the percentage of open area remaining, calculated as:

Remaining wound area (%) = (Area T_n_/Area T_0_) × 100.

### 2.5. Enzyme-Linked Immunosorbent Assay (ELISA)

To assess how FA influences the secretory profile of HGFs, MMP-1, TIMP-1, and IL-6 levels in conditioned media were determined by enzyme-linked immunosorbent assay (ELISA). After the 72 h glucose pre-conditioning phase, cells were exposed to FA at 25 and 50 µM for 24, 48, and 72 h under the conditions already described. Conditioned media were harvested from the scratch-assay plates described in Section 2.4 at each time point. Media collected at every interval were clarified by centrifugation (1000× *g*, 5 min) to pellet debris and then archived at −80 °C pending analysis. The three analytes were quantified with commercial kits (Elabscience Biotechnology, Wuhan, China), processed as directed by the supplier.

In brief, 100 µL of each sample was dispensed into the pre-coated wells and held at 37 °C for 2 h. The liquid was subsequently aspirated without an intervening wash, after which 100 µL biotinylated detection antibody was introduced and left for a further 1 h at 37 °C. Following three rinses, 100 µL HRP-conjugated avidin was applied and incubated for 1 h at 37 °C. Wells were rinsed three more times, and 90 µL TMB substrate was then added. Color development proceeded for 10 min at 37 °C under light protection before being halted with 50 µL stop solution. Optical density was recorded at 450 nm on the same EnSpire 2300 Multilabel Plate Reader within 5 min, and analyte concentrations were interpolated from the respective standard curve.

### 2.6. Statistical Analysis

Each experimental condition was performed as three independent biological replicates (*n* = 3), defined as three separate cell cultures established on different days within a narrow passage range (passages 4–5), each independently pre-conditioned and treated. Technical replicates (three separate wells per group) within an experiment were averaged prior to analysis, and all statistical analyses were based on the independent biological replicates; results are reported as mean ± standard deviation. The measured variables (percentage cell viability and ELISA-derived secreted-protein concentrations) are continuous and were assumed a priori to be approximately normally distributed, consistent with their biological nature and with previous studies using the same assays; accordingly, a parametric analytical approach was pre-specified. The Shapiro–Wilk and Levene tests were applied only as supportive screening procedures and did not indicate significant departures from normality or homogeneity of variance. However, because each group comprised only three biological replicates, these tests are underpowered and were not used to establish the distribution. As separate plates were used for each time point, comparisons were performed within each time point using ordinary one-way ANOVA followed by Tukey’s multiple comparisons test. Non-parametric alternatives were not applied because, at this sample size, they provide even lower statistical power. The limited number of biological replicates and the reliance on an a priori parametric assumption are acknowledged as study limitations. Effect sizes are reported as R^2^ for each analysis. Statistical analyses, including assumption checks and inferential tests, and data visualization and graphical representation were performed using IBM SPSS Statistics (version 23; IBM Corp., Armonk, NY, USA) and GraphPad Prism (version 11.0.2; GraphPad Software, Boston, MA, USA). A *p*-value < 0.05 was considered statistically significant. For clarity, only selected comparisons are annotated in the figures; the complete statistical output (mean differences, 95% confidence intervals, and adjusted *p*-values for all pre-specified comparisons, together with the R^2^ value for each analysis) is provided in Supplementary Table S1.

## 3. Results

### 3.1. Cell Viability Assay

**Figure 1:**
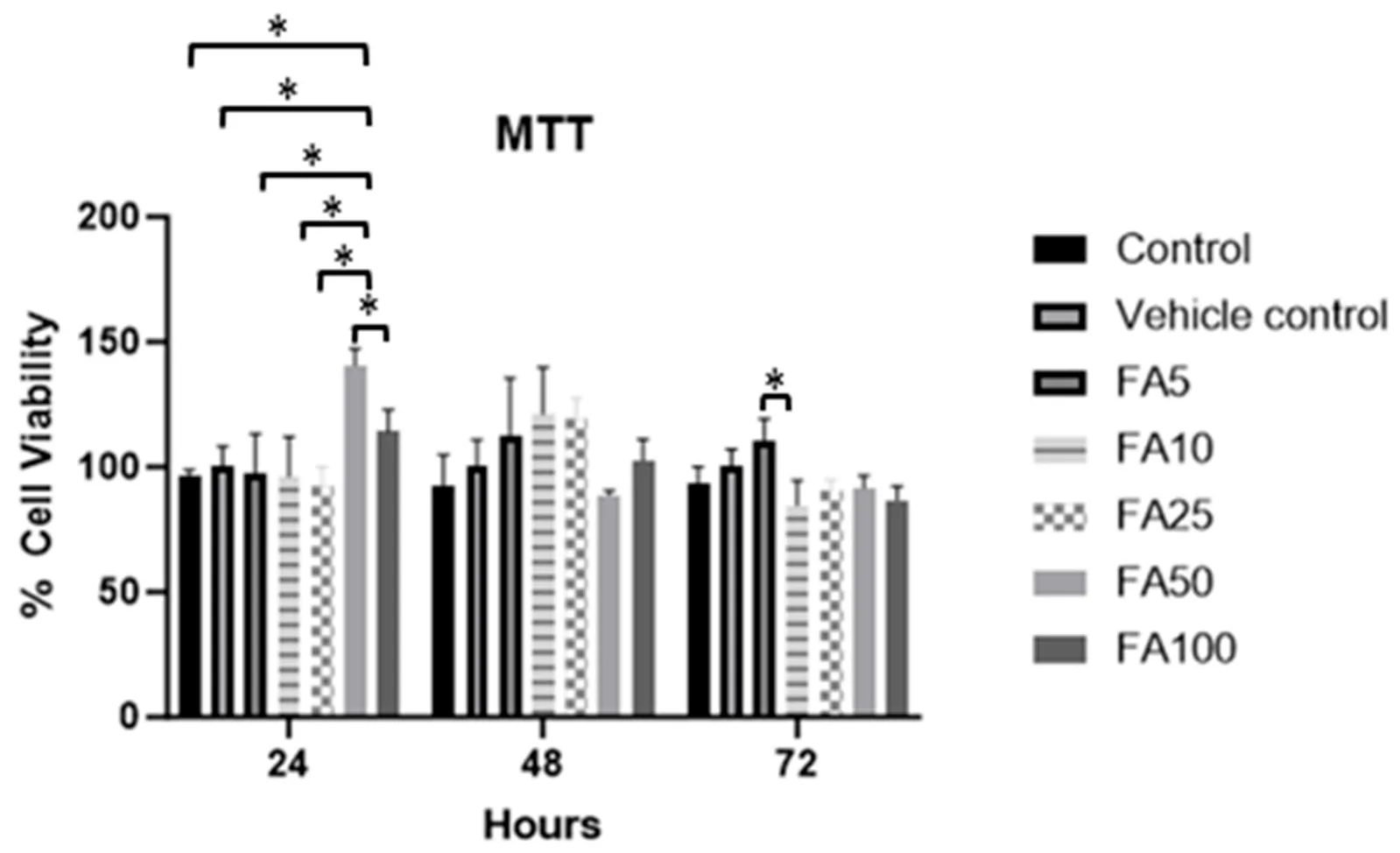
Effect of ferulic acid on HGF viability assessed by MTT assay at 24, 48, and 72 h. All results are expressed as percentages of survival compared to the vehicle control, which was set at 100%. Data are presented as mean ± SD of *n* = 3 independent biological replicates. Statistical analysis was performed using one-way ANOVA followed by Tukey’s HSD, * *p* < 0.05.

The viability of HGFs exposed to FA at 5, 10, 25, 50, and 100 µM, measured by the MTT test, is shown in Figure 1. None of the tested concentrations or time points produced a cytotoxic response, as every mean viability value stayed above the 70% non-cytotoxicity cut-off set by ISO 10993-5 [27].

Results are expressed as survival percentages relative to the vehicle control, which was normalized to 100%. Because separate wells were assigned to each time point, viability was compared among groups within each time point by ordinary one-way ANOVA followed by Tukey’s multiple comparisons test.

At 24 h, group comparisons revealed a statistically significant difference among groups (*p* = 0.001). The 50 µM FA group (140.78 ± 6.62%) showed significantly higher metabolic activity than all other groups (*p* < 0.05). At 48 h, no statistically significant differences were observed among the groups (*p* = 0.066). At 72 h, the 5 µM FA group exhibited significantly higher viability than the 10 µM group (*p* < 0.05), whereas no significant differences were detected between the 5 µM group and the remaining groups.

### 3.2. Wound Closure (Scratch) Assay

**Figure 2:**
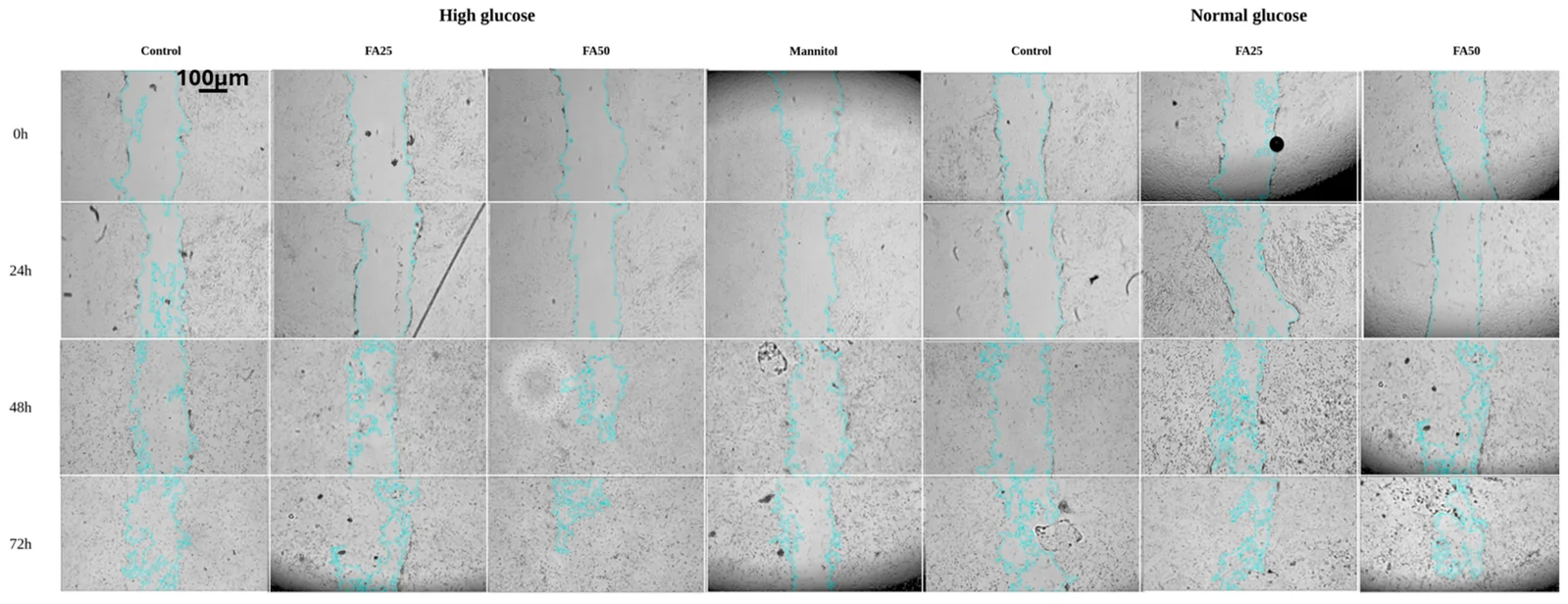
Cells were treated with ferulic acid (25 or 50 µM), with mannitol as an iso-osmolar control. Separate plates were used for each time point; representative images at 0, 24, 48, and 72 h are shown. Scale bar = 100 µm.

**Figure 3:**
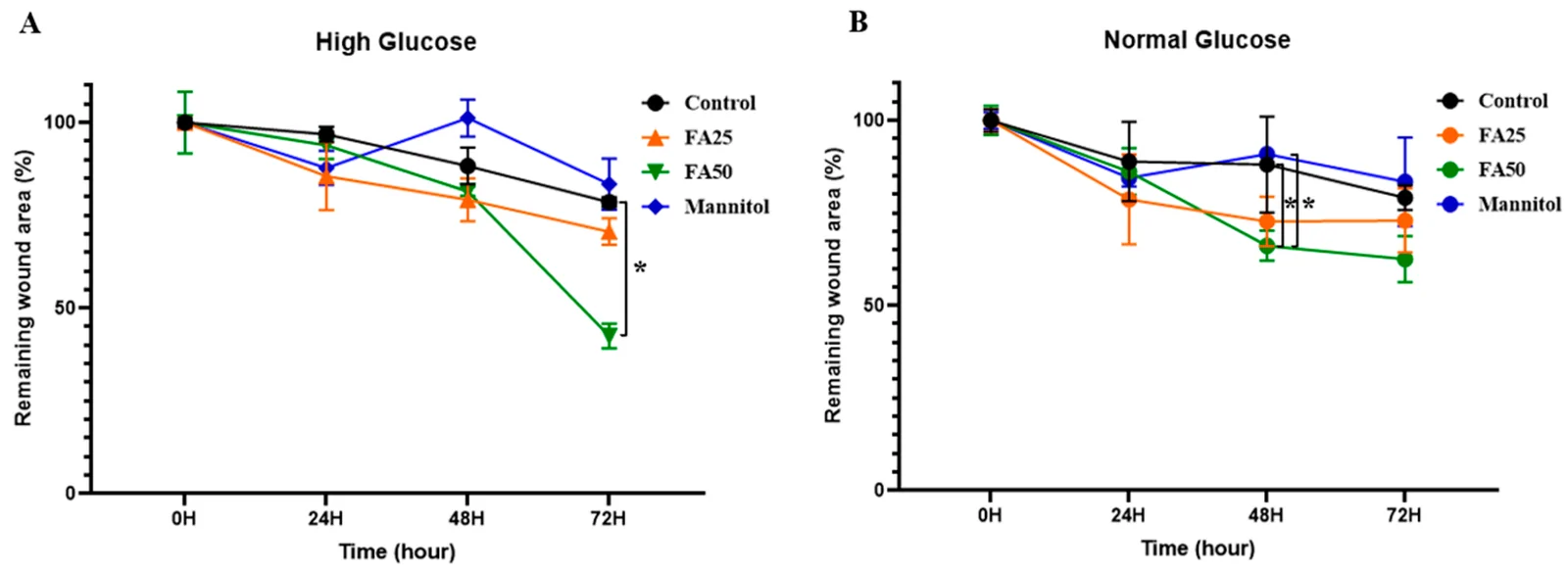
Effect of ferulic acid on the wound closure of human gingival fibroblasts (HGF) under high and normal glucose conditions. Confluent HGF monolayers were scratched and treated with FA at 25 or 50 μM, and the remaining wound area was measured at 0, 24, 48, and 72 h. (**A**) Remaining wound area (%) under high glucose conditions. (**B**) Remaining wound area (%) under normal glucose conditions. Data are presented as mean ± SD of *n* = 3 independent biological replicates. Statistical analysis was performed using ordinary one-way ANOVA followed by Tukey’s HSD; (* *p* < 0.05).

Following the scratch assay, serial images of FA-treated (25 and 50 μM) HGF cells were captured at baseline and at 24, 48, and 72-h intervals using an inverted light microscope (Figure 2), and wound closure rates were quantitatively determined by calculating the percentage reduction in scratch area with ImageJ software (Figure 3A,B). Under high glucose conditions (Figure 3A), wound closure progressed in all groups over time, yet FA accelerated this process most strongly at 50 μM. The FA50 group attained the lowest remaining wound area at 72 h, and this reduction was statistically significant relative to the untreated control (* *p* < 0.05). A comparable pattern emerged under normal glucose conditions (Figure 3B), where FA50 significantly reduced the remaining wound area compared with the untreated control at 48 h (* *p* < 0.05). The iso-osmolar mannitol control did not differ significantly from the untreated control at any time point under either glucose condition (*p* > 0.05), indicating that the osmolarity of the high-glucose medium did not by itself affect wound closure. At the two time points where FA50 significantly reduced the remaining wound area relative to the untreated control, it also differed significantly from the mannitol control (48 h under normal glucose, *p* < 0.05; 72 h under high glucose, *p* < 0.05), indicating that the improved closure reflected the activity of FA rather than an osmotic effect. Overall, FA enhanced wound closure (reflecting combined migration and proliferation) under both normal and high glucose conditions, with the effect confined to the 50 μM concentration. Under high-glucose conditions, HGF-1 cultures did not display overt morphological signs of stress: cells remained adherent with their characteristic spindle-shaped morphology, without evident rounding, detachment, or vacuolation relative to the normal-glucose control, consistent with viability values remaining above the 70% threshold. As no overt high-glucose–induced morphological change was apparent, a distinct corrective effect of FA on morphology could not be discerned; FA treatment was likewise not associated with any adverse morphological change.

### 3.3. Biochemical Findings

HGFs were treated with FA at concentrations of 25 and 50 µM for 24, 48, and 72 h, after which the concentrations of MMP-1, TIMP-1 and IL-6 were quantified in conditioned media using ELISA.

**Figure 4:**
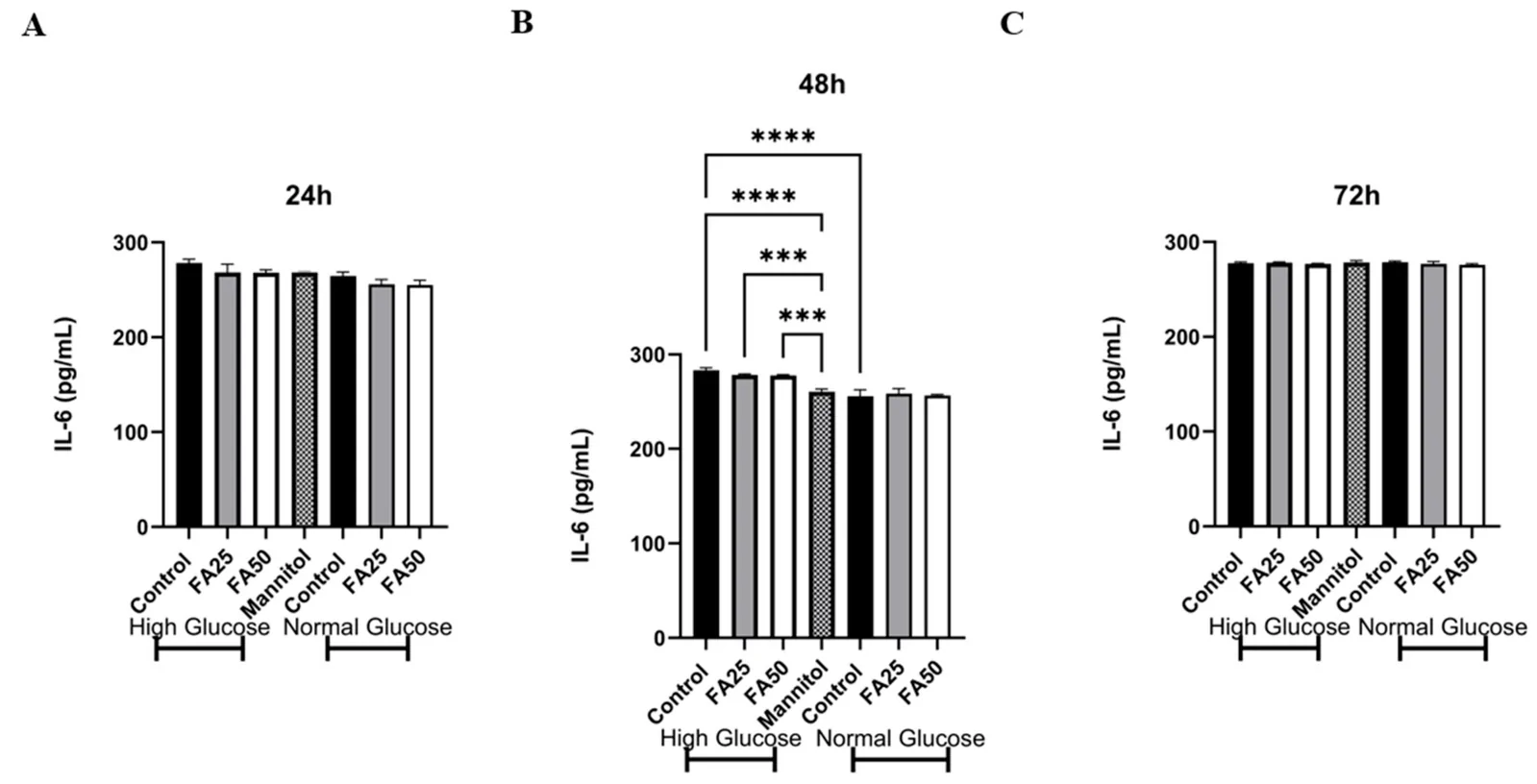
Effect of FA on IL-6 concentration by human gingival fibroblasts under high- and normal-glucose conditions. IL-6 levels (pg/mL) were measured by ELISA at (**A**) 24 h, (**B**) 48 h, and (**C**) 72 h. Cells were cultured under high glucose (HG) or normal glucose (NG), with mannitol included as an iso-osmolar mannitol control, and treated with ferulic acid (25 and 50 µM). Data are presented as mean ± SD (*n* = 3 independent biological replicates). Statistical analysis was performed using one-way ANOVA followed by Tukey’s HSD; *** *p* < 0.001, **** *p* < 0.0001.

At 24 h, IL-6 concentration showed a trend toward higher values in the high-glucose (HG) group compared with both the normal-glucose (NG) and iso-osmolar mannitol control groups; however, these differences did not reach statistical significance (*p* > 0.05) (Figure 4A). Treatment with FA resulted in a modest reduction in IL-6 levels under HG conditions, although this effect was not significant. By 48 h, the HG-induced increase in IL-6 became statistically significant, with IL-6 concentrations exceeding those observed in both the NG and iso-osmolar mannitol control (*p* < 0.0001) (Figure 4B). The absence of a comparable increase in the mannitol control indicated that the elevation of IL-6 concentration was attributable to glucose exposure rather than osmotic stress. FA produced only a minor attenuation of HG-induced IL-6 concentration at this time point; FA-treated HG groups remained significantly above the mannitol control (*p* < 0.001 for both), consistent with the absence of a significant FA effect on IL-6. In contrast, by 72 h, IL-6 levels had converged across all experimental groups, and no significant differences were detected (*p* > 0.05) (Figure 4C). Overall, high glucose induced a transient, glucose-specific increase in IL-6 concentration that reached significance at 48 h and resolved by 72 h, whereas FA exerted only a limited modulatory effect on the IL-6 response throughout the experimental period.

**Figure 5:**
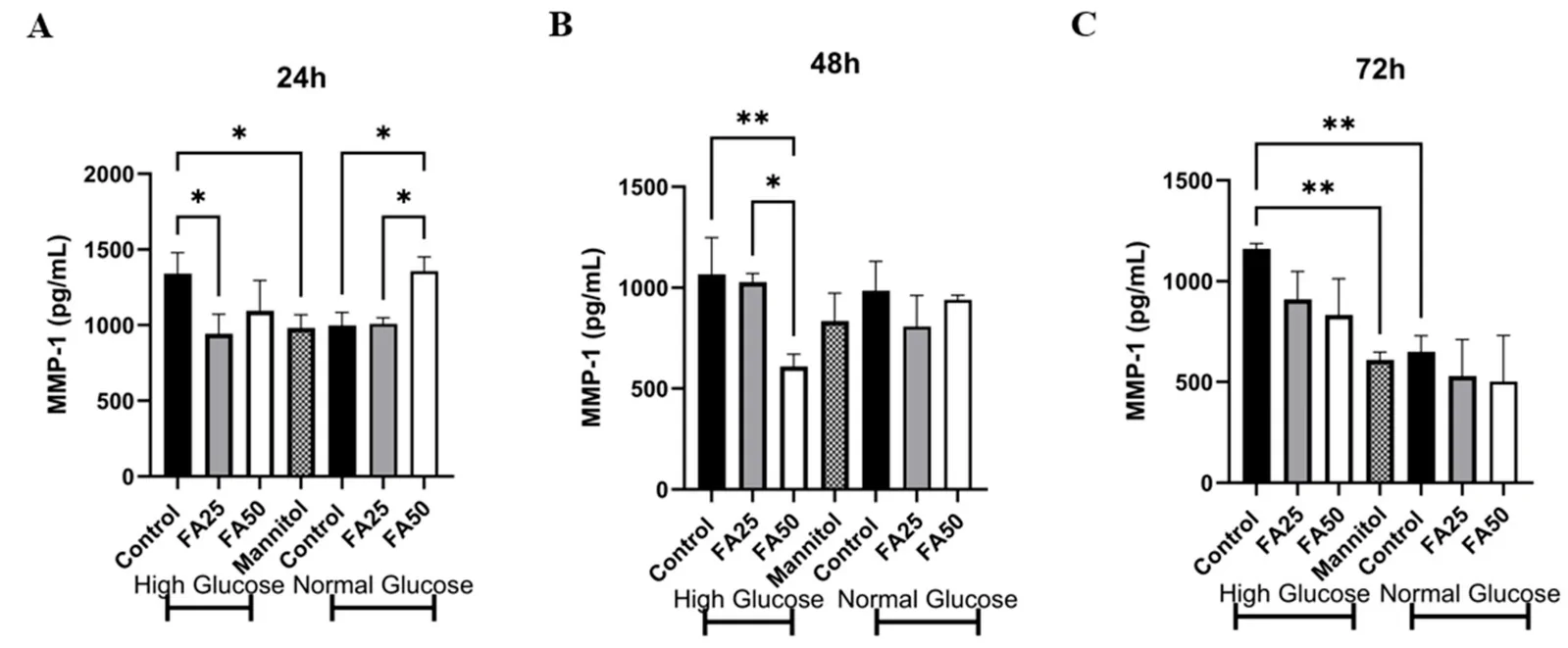
Effect of FA on MMP-1 concentration by human gingival fibroblasts under high- and normal-glucose conditions. MMP-1 levels (pg/mL) were measured by ELISA at (**A**) 24 h, (**B**) 48 h, and (**C**) 72 h. Cells were cultured under high glucose (HG) or normal glucose (NG), with mannitol included as an iso-osmolar mannitol control and treated with FA (25 and 50 µM). Data are presented as mean ± SD (*n* = 3 independent biological replicates). Statistical analysis was performed using one-way ANOVA followed by Tukey’s HSD; * *p* < 0.05, ** *p* < 0.01.

At 24 h, MMP-1 concentration under high glucose (HG) was significantly higher than in the iso-osmolar mannitol control (*p* < 0.05); levels were also higher than under normal glucose (NG), although this difference did not reach significance. FA reduced HG-induced MMP-1, with the reduction reaching significance at 25 µM (*p* < 0.05) (Figure 5A). At 48 h, MMP-1 levels did not differ significantly between the HG, NG and mannitol control groups, and the significant changes at this time point were confined to the HG group, where FA lowered MMP-1 at 50 µM (*p* < 0.01 vs. HG Control; *p* < 0.05 vs. HG FA25) (Figure 5B). By 72 h, the glucose-specific induction was most evident: MMP-1 under high glucose significantly exceeded both the mannitol control and the NG group (*p* < 0.01 for both), confirming that the increase was driven by glucose rather than osmolarity (Figure 5C). Overall, high glucose increased MMP-1 concentration in HGFs, an effect that was glucose-specific and statistically significant at 24 and 72 h, while FA reduced MMP-1 levels at 24 and 48 h, although the effective concentration was not consistent across time points.

**Figure 6:**
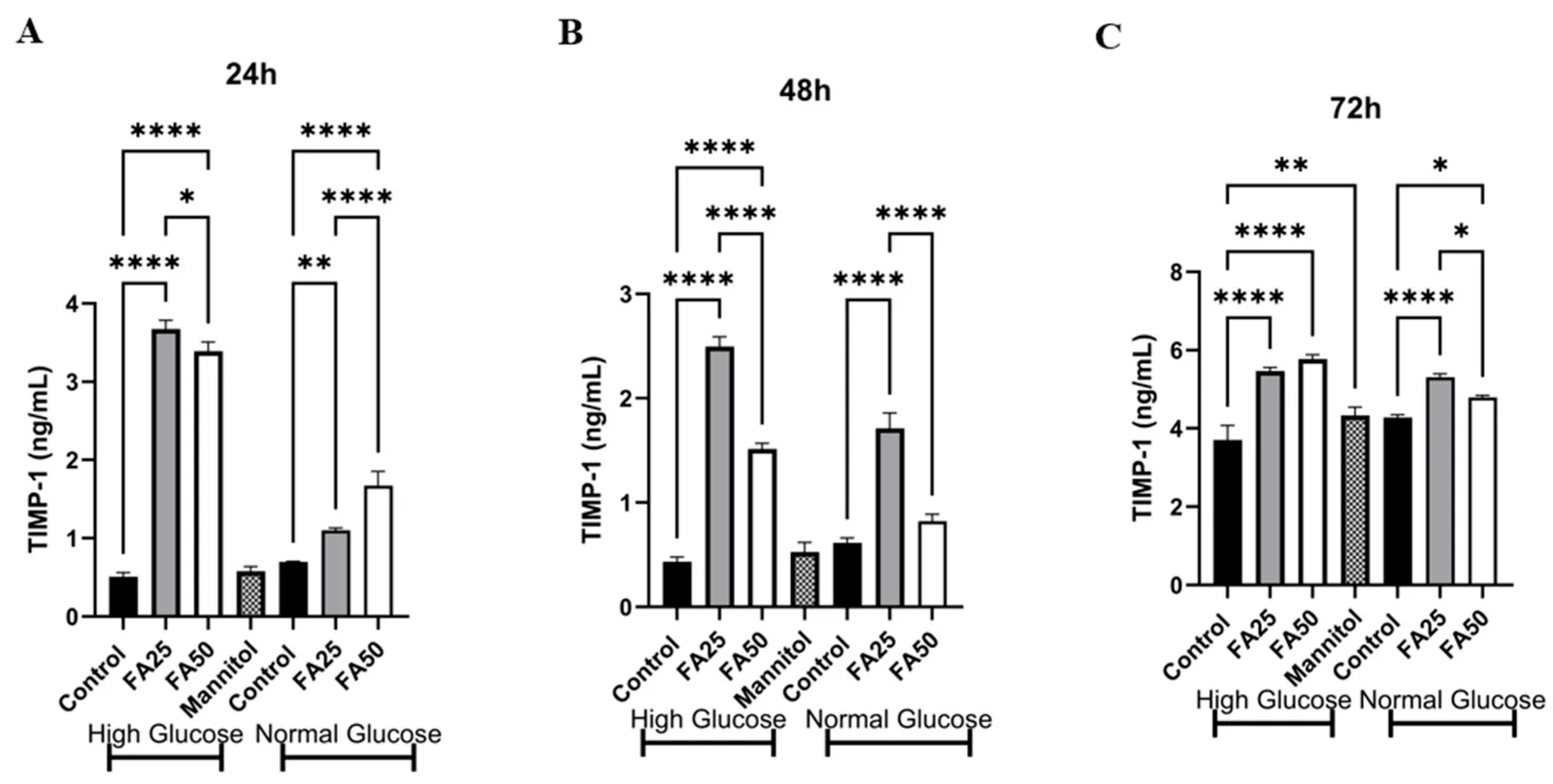
Effect of FA on TIMP-1 concentration by human gingival fibroblasts under high and normal-glucose conditions. TIMP-1 levels (ng/mL) were measured by ELISA at (**A**) 24 h, (**B**) 48 h, and (**C**) 72 h. Cells were cultured under high glucose (HG) or normal glucose (NG), with mannitol included as an iso-osmolar mannitol control and treated with FA (25 and 50 µM). Data are presented as mean ± SD (*n* = 3 independent biological replicates). Statistical analysis was performed using one-way ANOVA followed by Tukey’s HSD; * *p* < 0.05, ** *p* < 0.01, **** *p* < 0.0001.

TIMP-1 levels did not differ significantly between the untreated HG, untreated NG and iso-osmolar mannitol controls at 24 or 48 h (*p* > 0.05). By 72 h, however, TIMP-1 in the HG group was significantly lower than in both the NG control (*p* < 0.05) and the mannitol control (*p* < 0.01), whereas the NG and mannitol controls remained comparable, indicating a glucose-specific suppression of TIMP-1 at this time point (Figure 6A–C). In contrast, FA markedly increased TIMP-1 levels compared with untreated controls. Under high glucose, this increase was significant at both concentrations at all three time points (*p* < 0.0001 throughout). Under normal glucose, it was significant at 24 h (25 µM, *p* < 0.01; 50 µM, *p* < 0.0001) and at 72 h (25 µM, *p* < 0.0001; 50 µM, *p* < 0.05), whereas at 48 h only 25 µM reached significance (*p* < 0.0001). The two FA concentrations differed significantly at most time points, but not in a consistent direction. Overall, FA strongly enhanced TIMP-1 concentration under both glucose conditions, with a consistently larger effect under high glucose.

**Figure 7:**
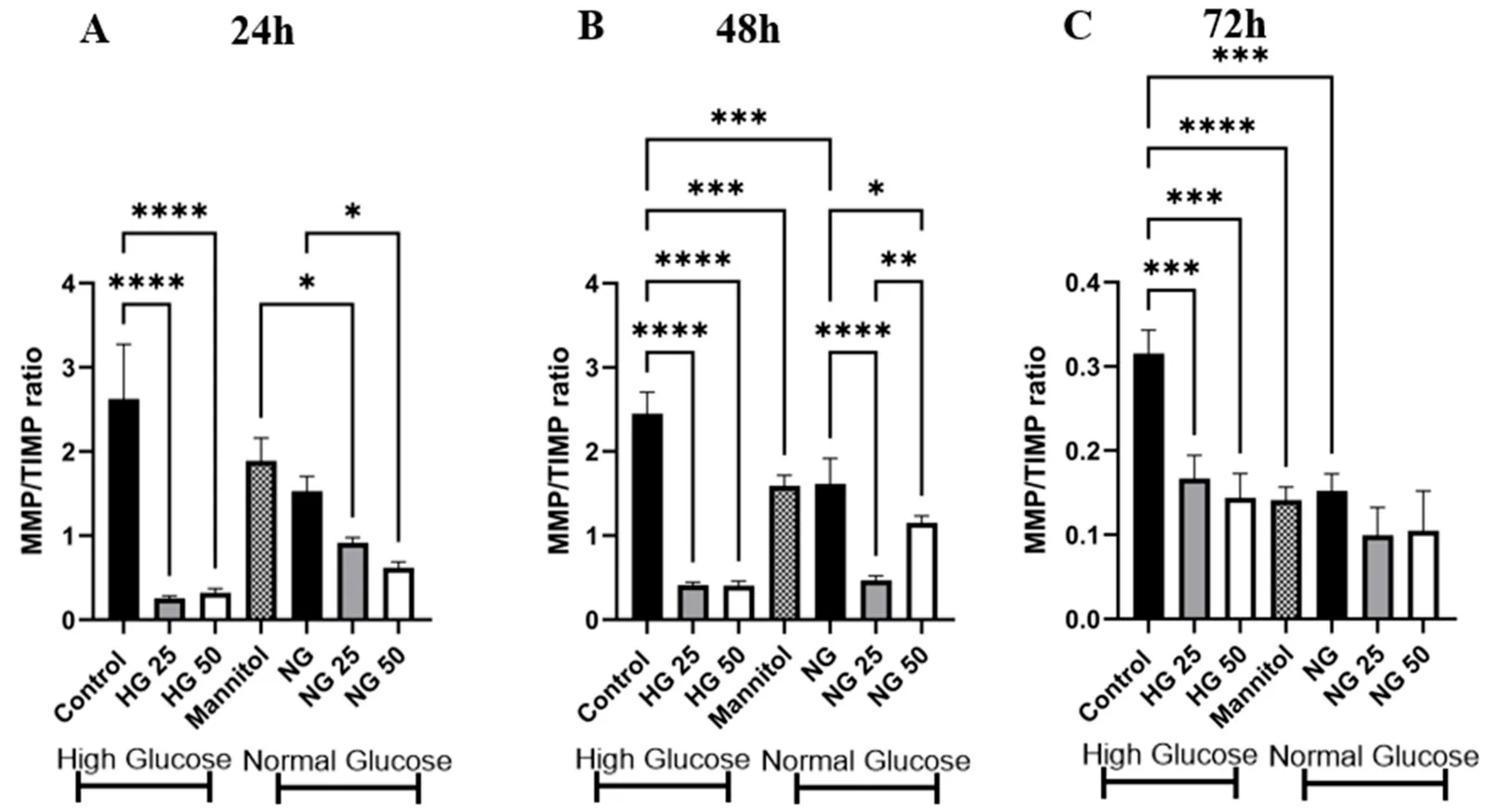
Effect of ferulic acid on the MMP-1/TIMP-1 ratio in human gingival fibroblasts under high and normal-glucose conditions. The ratio was calculated from unit-matched ELISA concentrations (MMP-1 in pg/mL divided by TIMP-1 converted to pg/mL) at (**A**) 24 h, (**B**) 48 h, and (**C**) 72 h. Data are presented as mean ± SD (*n* = 3 independent biological replicates). Statistical analysis was performed using one-way ANOVA followed by Tukey’s HSD (* *p* < 0.05, ** *p* < 0.01, *** *p* < 0.001, **** *p* < 0.0001).

To integrate the combined effects of FA on matrix-remodeling regulation, the MMP-1/TIMP-1 concentration ratio was calculated for each experimental group using unit-matched concentrations (Figure 7). At 24 h, the high glucose control exhibited the highest mean MMP-1/TIMP-1 ratio among the experimental groups, significantly exceeding the normal glucose control (*p* < 0.01), although the difference from the iso-osmolar mannitol control did not reach significance (*p* = 0.069). FA markedly reduced the ratio under high glucose at both concentrations (*p* < 0.0001 for both), whereas under normal glucose only 50 µM produced a significant reduction (*p* < 0.05) (Figure 7A).

At 48 h, the glucose-dependent shift was more clearly defined: the high glucose control exceeded both the normal glucose (*p* < 0.001) and mannitol (*p* < 0.001) controls, which did not differ from each other, indicating that the elevated ratio was attributable to glucose rather than osmolarity. FA reduced the ratio under high glucose at both concentrations (*p* < 0.0001 for both), and under normal glucose at 25 µM (*p* < 0.0001), while the reduction at 50 µM did not reach significance (*p* = 0.055) (Figure 7B).

At 72 h, overall MMP-1/TIMP-1 ratios decreased across all groups, coinciding with increased TIMP-1 levels; nevertheless, the glucose-specific elevation persisted, with the high glucose control exceeding both the normal glucose (*p* < 0.001) and mannitol (*p* < 0.0001) controls. FA restored the ratio under high glucose to values comparable with the mannitol and normal glucose controls (25 µM, *p* < 0.001; 50 µM, *p* < 0.0001 vs. high glucose control), whereas no significant effect of FA was detected under normal glucose (Figure 7C).

## 4. Discussion

Ferulic acid has drawn mounting attention as a potential therapeutic for inflammatory and metabolic disease. Across diverse model systems, it has been repeatedly shown to curb pro-inflammatory cytokine release, temper oxidative stress, and modulate the turnover of the ECM [22,28]. However, its potential utility in periodontal tissue biology under hyperglycemic conditions remains largely unexplored. The present study therefore sought to characterize the effects of FA on HGF viability, wound closure, and the secreted concentrations of MMP-1, TIMP-1, and IL-6 under normoglycemic and hyperglycemic conditions. FA did not exhibit cytotoxic effects on HGFs at the concentrations tested, indicating that it can be used safely in this cell type. Furthermore, FA differentially modulated the secreted concentrations of MMP-1, TIMP-1, and IL-6 in a time-dependent manner: most notably, it reduced MMP-1 levels and markedly increased TIMP-1 levels, thereby shifting the MMP-1/TIMP-1 balance toward matrix preservation, whereas its effect on IL-6 concentration was modest and did not reach statistical significance. These findings suggest that FA may help restore the proteolytic balance disrupted by chronic hyperglycemia in the periodontal microenvironment, while exerting only a limited modulatory effect on the inflammatory response under the conditions tested. As only a single cytokine (IL-6) was assessed and its modulation by FA was modest and non-significant, no conclusion regarding a broader anti-inflammatory effect can be drawn; a wider panel including TNF-α, IL-1β, IL-8, and MCP-1 is required to characterize this aspect. To the best of our knowledge, this is the first study to examine the biological effects of FA in HGFs exposed to high-glucose conditions, addressing a gap in the current understanding of potential phytotherapeutic strategies for periodontal tissue repair under hyperglycemic conditions.

The choice of HGFs as the experimental model reflects the clinical reality that periodontal complications in diabetes originate within the gingival connective tissue itself. Although *in vitro* studies on phytochemicals frequently rely on dermal or immortalized cell lines, such models fail to capture the cellular microenvironment in which periodontal healing actually occurs. HGFs uniquely integrate pro-inflammatory signalling with ECM remodeling, positioning them as a physiologically relevant target for compounds intended to modulate gingival repair [29]. This relevance is reinforced by recent evidence demonstrating that MMP-1, MMP-2, MMP-9 and MMP-13 produced by gingival fibroblasts are central drivers of ECM degradation in periodontitis, with their expression positively correlated with disease severity [30]. By testing FA in this system under hyperglycemic conditions, the present findings may have translational relevance and warrant further investigation as a basis for adjunctive periodontal strategies in diabetic populations.

The MTT findings revealed that FA did not produce a cytotoxic effect on HGFs at any of the tested concentrations (5–100 µM) or time points, as all mean viability values remained well above the 70% threshold defined by ISO 10993-5. This observation is in line with the literature describing FA as a low-toxicity phenolic phytochemical with minimal cytotoxicity across multiple fibroblast lines within micromolar ranges [22,31]. At 24 h, treatment with 50 µM FA significantly enhanced HGF viability compared with all other experimental groups; however, this effect was not sustained at higher concentrations or after prolonged exposure. Such a response may be consistent with a hormetic-like dose–response pattern of FA, where low concentrations promote cell viability, whereas higher doses or prolonged exposure attenuate this effect [32,33]. At 72 h, this pattern shifted: the lowest concentration tested (5 µM) showed significantly higher viability than the 10 µM group (*p* < 0.05), whereas no significant differences were detected relative to the remaining groups. This inverse concentration relationship may be explained by the limited stability and potential degradation of FA under cell culture conditions, whereby lower initial concentrations may maintain bioactive levels for a longer duration, while higher concentrations may fall below the effective threshold prior to the 72 h endpoint [31,34]. A comparable concentration-dependent response has previously been reported by San Miguel et al. in HGFs treated with FA-containing polyphenol mixtures [35]. Taken together, our findings indicate that FA exhibits a favorable *in vitro* non-cytotoxicity profile in HGFs, while inducing a biphasic and time-dependent metabolic response, with maximal effects observed at 50 µM after 24 h and at 5 µM after 72 h. It should be noted that the MTT assay measures mitochondrial dehydrogenase activity rather than cell number directly; because hyperglycemia can perturb mitochondrial metabolism independently of viability, MTT signals are best interpreted as metabolic activity. In the present study, this concern is mitigated by conducting the viability screen under normoglycemic DMEM (Section 2.3), but it remains relevant to the interpretation of metabolic responses. As these are *in vitro* metabolic activity data, they indicate non-cytotoxicity according to ISO 10993-5 rather than biocompatibility or therapeutic safety [20]. Establishing biocompatibility and translational relevance would require *in vivo* studies assessing gingival healing and tissue toxicity, and these findings should therefore be regarded as preliminary.

A further consideration concerns mitochondrial function. Mitochondrial dysfunction is recognized as a central, and often underappreciated, contributor to the pathogenesis of diabetes and its complications [36]. Because hyperglycemia impairs mitochondrial bioenergetics and increases reactive oxygen species production, gingival fibroblasts in the diabetic periodontium are exposed to sustained metabolic stress that compromises their proliferative and matrix-synthetic capacity. FA has been shown to counteract these processes, attenuating diabetes-linked complications and improving metabolic health in models of obesity-induced metabolic dysfunction [37]. This activity bears directly on the interpretation of our MTT data: because the assay reports mitochondrial dehydrogenase activity, the increased formazan signal at 25 and 50 µM FA may reflect improved mitochondrial function in addition to, or rather than, an increase in cell number alone. This interpretation was not tested experimentally, and dedicated assessment of mitochondrial endpoints in hyperglycemic HGFs represents an important direction for future work.

In addition to its favorable safety profile, FA enhanced wound closure in the scratch assay under both normal- and high-glucose conditions. Although wound closure increased progressively over time in all groups, FA-treated cells consistently exhibited a smaller residual wound area, with the most pronounced effect observed at 50 µM. Compared with the untreated control, this improvement reached statistical significance at 48 h under normal-glucose conditions and at 72 h under high-glucose conditions. Notably, the iso-osmolar mannitol control displayed a wound closure rate comparable to that of the untreated control and remained less effective than the FA-treated groups throughout the experiment. This indicates that the enhanced wound closure was attributable to a specific biological effect of FA rather than to the increased osmolarity of the high-glucose medium. This distinction is particularly relevant because hyperglycaemia is known to impair HGF proliferation and migration and to delay gingival wound healing through glucose-induced oxidative stress rather than osmotic stress [9]. Therefore, the ability of FA to promote wound closure under hyperglycaemic conditions suggests its potential to counteract key cellular mechanisms underlying diabetes-associated impairments in gingival healing. Because the scratch assay was performed without proliferation inhibition, part of this enhanced closure is likely attributable to increased proliferation rather than migration alone; this is consistent with the transient ~40% increase in metabolic activity observed at 50 µM after 24 h in the MTT assay.

The enhanced wound closure observed here is consistent with the well-established wound-healing properties of FA, which accelerates re-epithelialisation in streptozotocin-induced diabetic rats [38], improves healing in FA-loaded diabetic wound systems [39], and enhances human oral fibroblast responses *in vitro* [35]. Using the same *in vitro* model, we previously showed that another plant-derived compound promoted HGF wound closure [26], supporting the utility of this system for screening phytochemicals in gingival wound repair. The present findings extend these observations, at the level of wound closure, to HGFs exposed to hyperglycaemic stress and together with the concomitant reduction in MMP-1 levels and increase in TIMP-1 levels described below, suggest that the enhanced wound closure is associated with a shift towards a more matrix-protective phenotype. In this *in vitro* model, this combined effect on wound closure and the secreted MMP-1/TIMP-1 balance may be relevant to the diabetic periodontium, where impaired fibroblast activity and excessive matrix degradation contribute to delayed healing [40]; this link, however, remains to be confirmed in primary and diabetic fibroblasts and *in vivo*. These observations nonetheless indicate that FA warrants further investigation as a potential adjunctive agent in periodontal wound management.

In the present study, high glucose increased IL-6 concentration in the conditioned medium of HGFs, and the inclusion of an iso-osmolar mannitol control indicated that this response was attributable to glucose-specific signalling rather than a non-specific consequence of increased osmolarity. This induction reached statistical significance at 48 h, when IL-6 under high glucose exceeded both the normal-glucose and mannitol controls, supporting the concept that hyperglycaemia promotes a pro-inflammatory fibroblast phenotype. These findings are consistent with previous evidence showing that elevated glucose enhances inflammatory responses in gingival fibroblasts and contributes to periodontal inflammation under diabetic conditions [16,41].

Although FA is reported to decrease inflammatory mediators such as IL-6, TNF-α and IL-1β [42,43], its effect on IL-6 in the present model was limited and non-significant. The glucose-specific IL-6 increase was transient, reaching significance at 48 h and disappearing by 72 h. Thus, high glucose triggered an early but temporary IL-6 response in HGFs, whereas FA’s modulation of this marker was modest; a more pronounced effect was observed for the matrix-remodelling markers (MMP-1 and TIMP-1), discussed below.

High glucose increased MMP-1 concentration in the conditioned medium, and the inclusion of an iso-osmolar mannitol control demonstrated that this response was attributable to glucose exposure rather than increased medium osmolarity. Under high-glucose conditions, MMP-1 levels were elevated compared with the mannitol control at 24 h and became significantly higher than both normal-glucose and mannitol groups at 72 h, indicating a glucose-specific induction that was most evident at the later time point. These findings are consistent with the established role of hyperglycaemia in promoting ECM degradation and matrix-remodelling activity in periodontal tissues. Furthermore, hyperglycaemic exposure has been reported to enhance MMP-1 production in HGFs, providing a mechanistic explanation for the increased susceptibility to periodontal tissue breakdown associated with diabetes [41]. More broadly, hyperglycaemia may drive fibroblasts towards a more catabolic phenotype characterized by enhanced matrix metalloproteinase activity and impaired ECM homeostasis [40]. This response is further supported by evidence implicating NF-κB signalling as a key upstream regulator of MMP-1 expression under inflammatory and metabolic stress conditions [44]. Overall, these results strongly support the glucose-specific induction of MMP-1 observed in the present study and reinforce the relevance of HGFs as an *in vitro* model for investigating diabetes-associated periodontal matrix remodelling.

FA attenuated the high glucose-induced MMP-1 response, consistent with its reported ability to downregulate MMP-1/MMP-3 and to modulate the MMP/TIMP balance in fibroblasts [43,45,46]. The reduction in MMP-1 levels in FA-treated high-glucose groups aligns with this literature and, together with the cytokine results, indicates that FA may attenuate both inflammatory signalling and matrix-degrading responses associated with hyperglycaemic fibroblast activation. Notably, the relative effects of the two FA concentrations varied between time points, with the lower dose showing a greater effect at 24 h and the higher dose demonstrating a more pronounced reduction at 48 h. Although this pattern might tentatively be attributed to the biphasic or hormetic dose–response behaviour reported for FA and other polyphenolic compounds [32,33], this interpretation remains speculative: the present two-concentration design cannot establish a formal dose–response relationship, and the effects varied across time points. Dedicated dose–response studies with additional concentrations and mechanistic analyses are therefore required to clarify the underlying basis of this response.

TIMP-1 is an inducible member of the tissue inhibitor of metalloproteinases family and serves as a major endogenous regulator of MMP activity. Its expression can be upregulated by several inflammatory and regulatory mediators, including IL-1β, TGF-β1, retinoids, and IL-6, and disruption of MMP/TIMP homeostasis contributes to a variety of inflammatory and ECM remodelling disorders [47]. Functionally, the overall outcome of ECM turnover is determined by the relative balance between MMPs and TIMPs: a shift toward TIMP activity preserves matrix integrity and limits proteolysis, whereas predominance of MMP activity promotes matrix degradation [48]. Since the fibrillar collagenase MMP-1 is directly inhibited by TIMP-1, the MMP-1/TIMP-1 ratio represents a particularly relevant indicator of collagen turnover and matrix stability [49].

The principal finding for TIMP-1 was that FA increased its levels under both glucose conditions, which is consistent with previous reports describing its regulatory effects on the MMP/TIMP system. A FA-containing formulation has been shown to modulate MMP/TIMP signalling by reducing MMP-2/-9 activity while increasing TIMP-1 expression [50]. Similarly, FA alone has been reported to decrease MMP-3 and MMP-9 expression while enhancing TIMP-1 expression at the gene level [51]. When considered together with the observed reduction in MMP-1 levels, the increase in TIMP-1 levels suggests that FA shifts the MMP-1/TIMP-1 axis towards a more matrix-protective secretory profile at the protein level. As these are secreted-protein measurements, they do not establish net collagenolytic activity or verified enzyme–inhibitor engagement; the combined reduction in MMP-1 levels and increase in TIMP-1 levels may nonetheless favour ECM preservation more than changes in either component alone.

The opposing modulation of MMP-1 and TIMP-1 concentrations by FA prompted us to examine their ratio, which provides a more integrated measure of ECM turnover than either marker alone, as the balance between collagenolytic activity and endogenous inhibition determines whether matrix degradation or preservation predominates [49]. In our model, high glucose produced the highest MMP-1/TIMP-1 ratio, exceeding both the osmotic and normal-glucose controls, indicating a glucose-driven shift toward a catabolic, matrix-degrading state. This is consistent with periodontal evidence that the ratio rises as disease severity increases, reflecting the loss of inhibitory control over collagenolysis in inflamed gingival tissue [52]. FA reversed this shift, lowering the ratio under both glucose conditions through the combined effect of reduced MMP-1 and elevated TIMP-1 concentrations. A directly comparable change has been reported clinically, where successful phase I periodontal therapy decreased gingival crevicular fluid MMP-1, increased TIMP-1, and returned the MMP-1/TIMP-1 ratio toward that of healthy controls [52,53]. The convergence of group differences at 72 h suggests that FA-mediated rebalancing may be most evident during the early phase of fibroblast activation, when hyperglycemic stress exerts its strongest catabolic influence. Collectively, FA modulated the overall proteolytic equilibrium rather than targeting a single enzyme, promoting a more matrix-preserving profile that may be particularly relevant for impaired collagen homeostasis in the diabetic periodontium. It should be emphasized that antioxidant activity, the hormetic dose–response pattern, mitochondrial protection, and NF-κB modulation were drawn from the literature and were not evaluated experimentally in the present study.

This study has several limitations that should be considered when interpreting the findings. MMP-1, TIMP-1, and IL-6 were evaluated only at the secreted protein level, without assessment of gene expression or upstream signalling pathways, limiting the ability to define the molecular mechanisms underlying the observed effects. In addition, the study was performed entirely *in vitro* using a HGF model, which may not fully represent the biological complexity and cellular interactions of the periodontal microenvironment. In particular, the immortalized HGF-1 line was used to maximize standardization and comparability with established high-glucose gingival-fibroblast models; however, immortalized lines may not fully recapitulate primary gingival fibroblast behavior, donor-to-donor variability, or the diabetic cellular phenotype, and validation in primary and patient-derived diabetic fibroblasts is therefore warranted. Furthermore, the MMP-1/TIMP-1 ratio was calculated based on secreted protein concentrations rather than direct enzymatic activity measurements; therefore, it reflects relative protein abundance rather than actual collagenolytic capacity. Functional matrix-remodeling assays—gelatin/collagen zymography, an active MMP-1 activity assay, and a three-dimensional collagen gel contraction or collagen degradation/deposition readout—were not performed and are required to confirm the functional consequences of these changes; we note that gel contraction primarily reflects cellular contractility rather than collagenolysis and should be interpreted accordingly. In addition, the scratch assay was performed in complete medium (10% FBS) without pharmacological suppression of proliferation (e.g., mitomycin C), and no independent migration assay (e.g., transwell) was performed; consequently, wound closure reflects the combined effect of cell migration and proliferation rather than migration alone. Serum-reduced or serum-free scratch assays with fixed-field imaging, combined with an independent migration assay, are required to isolate the specific pro-migratory contribution of FA. A further limitation is that the 72 h high-glucose preconditioning models acute hyperglycemic exposure and does not reproduce chronic diabetic conditions, the accumulation of advanced glycation end-products, the chronic oxidative stress and persistent inflammation, or the long-term metabolic memory that characterize diabetic tissue, nor the microbial and inflammatory components involved in periodontal tissue destruction. The present findings should therefore be extrapolated to chronic diabetes with caution. Cell viability was assessed using a single metabolic assay (MTT), which reports mitochondrial dehydrogenase activity rather than cell number directly. In addition, cell morphology was assessed only qualitatively from the phase-contrast records of the scratch assay and was not systematically quantified; quantitative morphometric analysis (cell area, circularity, aspect ratio) on dedicated, standardized images represents an important direction for future work. Orthogonal confirmation with independent, non-metabolic methods, including direct cell counting, trypan blue exclusion, crystal violet staining, or Hoechst/propidium iodide double staining, was not performed in the present study and represents an important direction for future work, both to corroborate the non-cytotoxicity of FA and to determine whether the increased MTT signal at 25 and 50 µM reflects an increase in cell number rather than enhanced metabolic activity.

## 5. Conclusions

This study provides evidence that ferulic acid exerts beneficial *in vitro* effects on HGFs under the conditions tested. FA was non-cytotoxic across all tested concentrations (ISO 10993-5), and under high glucose, 50 µM FA significantly reduced the remaining wound area at 72 h, whereas no significant advantage over the untreated control was detected under normal glucose. At the molecular level, FA attenuated the high glucose-induced increase in MMP-1 concentration and increased TIMP-1 levels at most time points, shifting the secreted MMP-1/TIMP-1 concentration ratio toward a more favorable, matrix-protective profile at the protein level, whereas its effects on IL-6 concentration modulation were limited. These findings describe FA-induced changes in the secreted concentrations of MMP-1, TIMP-1, and IL-6 and in wound closure under metabolic stress. As such, they reflect altered protein secretion rather than directly demonstrated extracellular matrix remodeling, and provide a basis for further investigation of its relevance to periodontal tissue repair. As no signalling pathways were assessed, mechanistic interpretations are not drawn here; future *in vivo* and mechanistic studies are warranted to validate these findings and clarify their translational significance.

## Data Availability

The original contributions presented in this study are included in the article/Supplementary Materials. Further inquiries can be directed to the corresponding author.

## Supplementary Materials

The following supporting information can be downloaded at: https://www.mdpi.com/article/10.3390/biomedicines14081803/s1, Supplementary Table S1. Complete statistical output for all pre-specified comparisons. Mean differences, 95% confidence intervals, adjusted *p*-values, and corresponding effect sizes (R^2^) are reported for each analysis. This table provides the complete statistical results supporting the simplified statistical annotations presented in the main figures.

## Abbreviations

The following abbreviations are used in this manuscript:

DMEM	Dulbecco’s Modified Eagle’s Medium
DMSO	Dimethyl sulfoxide
ECM	Extracellular matrix
ELISA	Enzyme-linked immunosorbent assay
FA	Ferulic acid
FBS	Fetal bovine serum
HG	High glucose
HGF	Human gingival fibroblast
IL	Interleukin
ISO	International Organization for Standardization
MMP	Matrix metalloproteinase
MTT	3-(4,5-dimethylthiazol-2-yl)-2,5-diphenyltetrazolium bromide
NF-κB	Nuclear factor kappa B
NG	Normal glucose
PBS	Phosphate-buffered saline
SD	Standard deviation
TIMP	Tissue inhibitor of metalloproteinases
