# Supplementary material for: Ferulic Acid Modulates High Glucose-Induced MMP-1/TIMP-1 Imbalance and Enhances Wound Closure of Human Gingival Fibroblasts
Source: Biomedicines. 2026 Aug 11;14(8):1803. doi: 10.3390/biomedicines14081803 (PMC13509705; doi:10.3390/biomedicines14081803)
Supplement: Supplementary file 1 [file biomedicines-14-01803-s001.zip › biomedicines-4419141-supplementary.pdf]

Supplementary Table S1. Complete statistical output for all pre-specified comparisons. Mean differences, 95% confidence intervals, adjusted *p*-values, and corresponding effect sizes (R^2) are reported for each analysis. This table provides the complete statistical results supporting the simplified statistical annotations presented in the main figures.

IL-6, 24 h

R^2 = 0.7732; Tukey's multiple comparisons test; n = 3 independent biological replicates.

High-glucose groups

Comparison	Mean diff.	95% CI	Adjusted P	
HG control vs HG+FA25	9.981	−3.769 to 23.73	0.2380	ns
HG control vs HG+FA50	10.13	−3.621 to 23.88	0.2252	ns
HG control vs Mannitol	9.981	−3.769 to 23.73	0.2380	ns
HG+FA25 vs HG+FA50	0.1479	−13.60 to 13.90	> 0.9999	ns
HG+FA25 vs Mannitol	0.000	−13.75 to 13.75	> 0.9999	ns
HG+FA50 vs Mannitol	−0.1479	−13.90 to 13.60	> 0.9999	ns

Normal-glucose groups

Comparison	Mean diff.	95% CI	Adjusted P	
NG control vs NG+FA25	8.790	−4.959 to 22.54	0.3616	ns
NG control vs NG+FA50	9.409	−4.341 to 23.16	0.2929	ns
NG+FA25 vs NG+FA50	0.6183	−13.13 to 14.37	> 0.9999	ns
Mannitol vs NG control	3.696	−10.05 to 17.45	0.9633	ns
Mannitol vs NG+FA25	12.49	−1.263 to 26.24	0.0867	ns
Mannitol vs NG+FA50	13.10	−0.6449 to 26.85	0.0664	ns

Controls

Comparison	Mean diff.	95% CI	Adjusted P	
HG control vs NG control	13.68	−0.07234 to 27.43	0.0516	ns
HG control vs Mannitol	9.981	−3.769 to 23.73	0.2380	ns
Mannitol vs NG control	3.696	−10.05 to 17.45	0.9633	ns

IL-6, 48 h

R^2 = 0.9317; Tukey's multiple comparisons test; n = 3 independent biological replicates.

High-glucose groups

Comparison	Mean diff.	95% CI	Adjusted P	
HG control vs HG+FA25	4.818	−5.505 to 15.14	0.6884	ns
HG control vs HG+FA50	5.392	−4.930 to 15.72	0.5778	ns
HG control vs Mannitol	22.74	12.42 to 33.07	**< 0.0001**	****
HG+FA25 vs HG+FA50	0.5747	−9.748 to 10.90	> 0.9999	ns
HG+FA25 vs Mannitol	17.93	7.604 to 28.25	**0.0006**	***
HG+FA50 vs Mannitol	17.35	7.029 to 27.67	**0.0008**	***

Normal-glucose groups

Comparison	Mean diff.	95% CI	Adjusted P	
NG control vs NG+FA25	−2.847	−13.17 to 7.476	0.9587	ns
NG control vs NG+FA50	−0.7151	−11.04 to 9.608	> 0.9999	ns
NG+FA25 vs NG+FA50	2.132	−8.191 to 12.45	0.9900	ns
Mannitol vs NG control	4.927	−5.395 to 15.25	0.6675	ns
Mannitol vs NG+FA25	2.081	−8.242 to 12.40	0.9912	ns
Mannitol vs NG+FA50	4.212	−6.110 to 14.54	0.7967	ns

Controls

Comparison	Mean diff.	95% CI	Adjusted P	
HG control vs NG control	27.67	17.35 to 37.99	**< 0.0001**	****
HG control vs Mannitol	22.74	12.42 to 33.07	**< 0.0001**	****
Mannitol vs NG control	4.927	−5.395 to 15.25	0.6675	ns

IL-6, 72 h

$R^2 = 0.3434$; Tukey's multiple comparisons test; n = 3 independent biological replicates.

High-glucose groups

Comparison	Mean diff.	95% CI	Adjusted P	
HG control vs HG+FA25	−0.6049	−4.743 to 3.533	0.9985	ns
HG control vs HG+FA50	0.7366	−3.402 to 4.875	0.9954	ns
HG control vs Mannitol	−0.7070	−4.845 to 3.431	0.9963	ns
HG+FA25 vs HG+FA50	1.341	−2.797 to 5.480	0.9158	ns
HG+FA25 vs Mannitol	−0.1021	−4.240 to 4.036	> 0.9999	ns
HG+FA50 vs Mannitol	−1.444	−5.582 to 2.695	0.8864	ns

Normal-glucose groups

Comparison	Mean diff.	95% CI	Adjusted P	
NG control vs NG+FA25	1.613	−2.525 to 5.751	0.8271	ns
NG control vs NG+FA50	2.608	−1.531 to 6.746	0.3769	ns
NG+FA25 vs NG+FA50	0.9946	−3.144 to 5.133	0.9786	ns
Mannitol vs NG control	−0.2581	−4.396 to 3.880	> 0.9999	ns
Mannitol vs NG+FA25	1.355	−2.783 to 5.493	0.9122	ns
Mannitol vs NG+FA50	2.349	−1.789 to 6.488	0.4891	ns

Controls

Comparison	Mean diff.	95% CI	Adjusted P	
HG control vs NG control	−0.9651	−5.103 to 3.173	0.9815	ns
HG control vs Mannitol	−0.7070	−4.845 to 3.431	0.9963	ns
Mannitol vs NG control	−0.2581	−4.396 to 3.880	> 0.9999	ns

MMP-1, 24 h

R^2 = 0.7267; Tukey's multiple comparisons test; n = 3 independent biological replicates.

High-glucose groups

Comparison	Mean diff.	95% CI	Adjusted P	
HG control vs HG+FA25	398.2	60.45 to 735.9	**0.0166**	*
HG control vs HG+FA50	246.1	−91.66 to 583.8	0.2348	ns
HG control vs Mannitol	360.8	23.08 to 698.5	**0.0329**	*
HG+FA25 vs HG+FA50	−152.1	−489.8 to 185.6	0.7198	ns
HG+FA25 vs Mannitol	−37.37	−375.1 to 300.3	0.9997	ns
HG+FA50 vs Mannitol	114.7	−223.0 to 452.4	0.8978	ns

Normal-glucose groups

Comparison	Mean diff.	95% CI	Adjusted P	
NG control vs NG+FA25	−11.58	−349.3 to 326.1	> 0.9999	ns
NG control vs NG+FA50	−360.0	−697.7 to −22.29	**0.0334**	*
NG+FA25 vs NG+FA50	−348.4	−686.1 to −10.71	**0.0412**	*
Mannitol vs NG control	−16.84	−354.6 to 320.9	> 0.9999	ns
Mannitol vs NG+FA25	−28.42	−366.1 to 309.3	> 0.9999	ns
Mannitol vs NG+FA50	−376.8	−714.6 to −39.13	**0.0245**	*

Controls

Comparison	Mean diff.	95% CI	Adjusted P	
HG control vs NG control	343.9	6.238 to 681.7	0.0547	ns
HG control vs Mannitol	360.8	23.08 to 698.5	**0.0329**	*
Mannitol vs NG control	−16.84	−354.6 to 320.9	> 0.9999	ns

MMP-1, 48 h

One-way ANOVA, $R^2 = 0,6854$; Tukey's multiple comparisons test; n = 3 independent biological replicates.

High-glucose groups

Comparison	Mean diff.	95% CI	Adjusted P	
HG control vs HG+FA25	38.86	−300.3 to 378.0	0.9996	ns
HG control vs HG+FA50	457.4	118.2 to 796.5	**0.0058**	**
HG control vs Mannitol	232.2	−107.0 to 571.4	0.2925	ns
HG+FA25 vs HG+FA50	418.5	79.33 to 757.7	**0.0118**	*
HG+FA25 vs Mannitol	193.3	−145.8 to 532.5	0.4848	ns
HG+FA50 vs Mannitol	−225.2	−564.4 to 114.0	0.3228	ns

Normal-glucose groups

Comparison	Mean diff.	95% CI	Adjusted P	
NG control vs NG+FA25	178.9	−160.3 to 518.0	0.5680	ns
NG control vs NG+FA50	44.39	−294.8 to 383.6	0.9992	ns
NG+FA25 vs NG+FA50	−134.5	−473.7 to 204.7	0.8162	ns
Mannitol vs NG control	−150.8	−490.0 to 188.4	0.7309	ns
Mannitol vs NG+FA25	28.07	−311.1 to 367.3	> 0.9999	ns
Mannitol vs NG+FA50	−106.4	−445.6 to 232.8	0.9267	ns

Controls

Comparison	Mean diff.	95% CI	Adjusted P	
HG control vs NG control	81.40	−257.8 to 420.6	0.9787	ns
HG control vs Mannitol	232.2	−107.0 to 571.4	0.2925	ns
Mannitol vs NG control	−150.8	−490.0 to 188.4	0.7309	ns

MMP-1, 72 h

R^2 = 0.7800; Tukey's multiple comparisons test; n = 3 independent biological replicates.

High-glucose groups

Comparison	Mean diff.	95% CI	Adjusted P	
HG control vs HG+FA25	249.5	−150.9 to 649.8	0.3887	ns
HG control vs HG+FA50	327.6	−72.74 to 728.0	0.1451	ns
HG control vs Mannitol	551.6	151.2 to 951.9	**0.0048**	**
HG+FA25 vs HG+FA50	78.16	−322.2 to 478.5	0.9926	ns
HG+FA25 vs Mannitol	302.1	−98.26 to 702.5	0.2051	ns
HG+FA50 vs Mannitol	223.9	−176.4 to 624.3	0.5052	ns

Normal-glucose groups

Comparison	Mean diff.	95% CI	Adjusted P	
NG control vs NG+FA25	119.7	−280.6 to 520.1	0.9405	ns
NG control vs NG+FA50	147.9	−252.5 to 548.3	0.8581	ns
NG+FA25 vs NG+FA50	28.16	−372.2 to 428.5	> 0.9999	ns
Mannitol vs NG control	−40.53	−440.9 to 359.8	0.9998	ns
Mannitol vs NG+FA25	79.21	−321.2 to 479.6	0.9920	ns
Mannitol vs NG+FA50	107.4	−293.0 to 507.7	0.9637	ns

Controls

Comparison	Mean diff.	95% CI	Adjusted P	
HG control vs NG control	511.1	110.7 to 911.4	**0.0090**	**
HG control vs Mannitol	551.6	151.2 to 951.9	**0.0048**	**
Mannitol vs NG control	−40.53	−440.9 to 359.8	0.9998	ns

TIMP-1, 24 h

R^2 = 0.9959; Tukey's multiple comparisons test; n = 3 independent biological replicates.

High-glucose groups

Comparison	Mean diff.	95% CI	Adjusted P	
HG control vs HG+FA25	−3.167	−3.440 to −2.895	**< 0.0001**	****
HG control vs HG+FA50	−2.885	−3.158 to −2.612	**< 0.0001**	****
HG control vs Mannitol	−0.07011	−0.3429 to 0.2027	0.9704	ns
HG+FA25 vs HG+FA50	0.2825	0.009675 to 0.5553	**0.0403**	*
HG+FA25 vs Mannitol	3.097	2.825 to 3.370	**< 0.0001**	****
HG+FA50 vs Mannitol	2.815	2.542 to 3.088	**< 0.0001**	****

Normal-glucose groups

Comparison	Mean diff.	95% CI	Adjusted P	
NG control vs NG+FA25	−0.4052	−0.6780 to −0.1324	**0.0025**	**
NG control vs NG+FA50	−0.9732	−1.246 to −0.7004	**< 0.0001**	****
NG+FA25 vs NG+FA50	−0.5680	−0.8408 to −0.2952	**< 0.0001**	****
Mannitol vs NG control	−0.1196	−0.3925 to 0.1532	0.7421	ns
Mannitol vs NG+FA25	−0.5249	−0.7977 to −0.2521	**0.0002**	***
Mannitol vs NG+FA50	−1.093	−1.366 to −0.8201	**< 0.0001**	****

Controls

Comparison	Mean diff.	95% CI	Adjusted P	
HG control vs NG control	−0.1898	−0.4626 to 0.08306	0.2772	ns
HG control vs Mannitol	−0.07011	−0.3429 to 0.2027	0.9704	ns
Mannitol vs NG control	−0.1196	−0.3925 to 0.1532	0.7421	ns

TIMP-1, 48 h

$R^2 = 0.9905$; Tukey's multiple comparisons test; n = 3 independent biological replicates.

High-glucose groups

Comparison	Mean diff.	95% CI	Adjusted P	
HG control vs HG+FA25	−2.062	−2.300 to −1.823	**< 0.0001**	****
HG control vs HG+FA50	−1.079	−1.317 to −0.8403	**< 0.0001**	****
HG control vs Mannitol	−0.09260	−0.3310 to 0.1458	0.8292	ns
HG+FA25 vs HG+FA50	0.9829	0.7445 to 1.221	**< 0.0001**	****
HG+FA25 vs Mannitol	1.969	1.731 to 2.207	**< 0.0001**	****
HG+FA50 vs Mannitol	0.9861	0.7477 to 1.224	**< 0.0001**	****

Normal-glucose groups

Comparison	Mean diff.	95% CI	Adjusted P	
NG control vs NG+FA25	−1.096	−1.334 to −0.8575	**< 0.0001**	****
NG control vs NG+FA50	−0.2059	−0.4443 to 0.03252	0.1123	ns
NG+FA25 vs NG+FA50	0.8900	0.6516 to 1.128	**< 0.0001**	****
Mannitol vs NG control	−0.08776	−0.3262 to 0.1507	0.8600	ns
Mannitol vs NG+FA25	−1.184	−1.422 to −0.9452	**< 0.0001**	****
Mannitol vs NG+FA50	−0.2936	−0.5321 to −0.05524	**0.0119**	*

Controls

Comparison	Mean diff.	95% CI	Adjusted P	
HG control vs NG control	−0.1804	−0.4188 to 0.05805	0.2030	ns
HG control vs Mannitol	−0.09260	−0.3310 to 0.1458	0.8292	ns
Mannitol vs NG control	−0.08776	−0.3262 to 0.1507	0.8600	ns

TIMP-1, 72 h

R^2 = 0.9579; Tukey's multiple comparisons test; n = 3 independent biological replicates.

High-glucose groups

Comparison	Mean diff.	95% CI	Adjusted P	
HG control vs HG+FA25	−1.766	−2.262 to −1.270	**< 0.0001**	****
HG control vs HG+FA50	−2.075	−2.570 to −1.579	**< 0.0001**	****
HG control vs Mannitol	−0.6279	−1.124 to −0.1321	**0.0096**	**
HG+FA25 vs HG+FA50	−0.3089	−0.8046 to 0.1869	0.3888	ns
HG+FA25 vs Mannitol	1.138	0.6422 to 1.634	**< 0.0001**	****
HG+FA50 vs Mannitol	1.447	0.9511 to 1.943	**< 0.0001**	****

Normal-glucose groups

Comparison	Mean diff.	95% CI	Adjusted P	
NG control vs NG+FA25	−1.032	−1.528 to −0.5366	**< 0.0001**	****
NG control vs NG+FA50	−0.5120	−1.008 to −0.01621	**0.0410**	*
NG+FA25 vs NG+FA50	0.5204	0.02463 to 1.016	**0.0369**	*
Mannitol vs NG control	0.05329	−0.4425 to 0.5490	0.9997	ns
Mannitol vs NG+FA25	−0.9791	−1.475 to −0.4833	**0.0001**	***
Mannitol vs NG+FA50	−0.4587	−0.9544 to 0.03708	0.0784	ns

Controls

Comparison	Mean diff.	95% CI	Adjusted P	
HG control vs NG control	−0.5746	−1.070 to −0.07882	**0.0188**	*
HG control vs Mannitol	−0.6279	−1.124 to −0.1321	**0.0096**	**
Mannitol vs NG control	0.05329	−0.4425 to 0.5490	0.9997	ns

MMP-1/TIMP-1 ratio, 24 h

$R^2 = 0.9288$; Tukey's multiple comparisons test; n = 3 independent biological replicates.

High-glucose groups

Comparison	Mean diff.	95% CI	Adjusted P	
HG control vs HG+FA25	2.365	1.591 to 3.138	**< 0.0001**	****
HG control vs HG+FA50	2.303	1.530 to 3.077	**< 0.0001**	****
HG control vs Mannitol	0.7323	−0.04120 to 1.506	0.0689	ns
HG+FA25 vs HG+FA50	−0.06157	−0.8351 to 0.7120	> 0.9999	ns
HG+FA25 vs Mannitol	−1.633	−2.406 to −0.8590	**< 0.0001**	****
HG+FA50 vs Mannitol	−1.571	−2.345 to −0.7975	**0.0001**	***

Normal-glucose groups

Comparison	Mean diff.	95% CI	Adjusted P	
NG control vs NG+FA25	0.6167	−0.1569 to 1.390	0.1630	ns
NG control vs NG+FA50	0.9133	0.1398 to 1.687	**0.0164**	*
NG+FA25 vs NG+FA50	0.2967	−0.4769 to 1.070	0.8369	ns
Mannitol vs NG control	0.3577	−0.4159 to 1.131	0.6968	ns
Mannitol vs NG+FA25	0.9743	0.2008 to 1.748	**0.0100**	*
Mannitol vs NG+FA50	1.271	0.4975 to 2.045	**0.0010**	***

Controls

Comparison	Mean diff.	95% CI	Adjusted P	
HG control vs NG control	1.090	0.3165 to 1.864	**0.0040**	**
HG control vs Mannitol	0.7323	−0.04120 to 1.506	0.0689	ns
Mannitol vs NG control	0.3577	−0.4159 to 1.131	0.6968	ns

MMP-1/TIMP-1 ratio, 48 h

R^2 = 0.9645; Tukey's multiple comparisons test; n = 3 independent biological replicates.

High-glucose groups

Comparison	Mean diff.	95% CI	Adjusted P	
HG control vs HG+FA25	2.009	1.541 to 2.477	**< 0.0001**	****
HG control vs HG+FA50	2.043	1.575 to 2.511	**< 0.0001**	****
HG control vs Mannitol	0.8567	0.3887 to 1.325	**0.0003**	***
HG+FA25 vs HG+FA50	0.0347	−0.4333 to 0.5026	> 0.9999	ns
HG+FA25 vs Mannitol	−1.152	−1.620 to −0.6841	**< 0.0001**	****
HG+FA50 vs Mannitol	−1.187	−1.655 to −0.7187	**< 0.0001**	****

Normal-glucose groups

Comparison	Mean diff.	95% CI	Adjusted P	
NG control vs NG+FA25	1.113	0.6454 to 1.581	**< 0.0001**	****
NG control vs NG+FA50	0.4600	−0.0079 to 0.9279	0.0554	ns
NG+FA25 vs NG+FA50	−0.6533	−1.121 to −0.1854	**0.0043**	**
Mannitol vs NG control	−0.0233	−0.4913 to 0.4446	> 0.9999	ns
Mannitol vs NG+FA25	1.090	0.6221 to 1.558	**< 0.0001**	****
Mannitol vs NG+FA50	0.4367	−0.0313 to 0.9046	0.0747	ns

Controls

Comparison	Mean diff.	95% CI	Adjusted P	
HG control vs NG control	0.8333	0.3654 to 1.301	**0.0004**	***
HG control vs Mannitol	0.8567	0.3887 to 1.325	**0.0003**	***
Mannitol vs NG control	−0.0233	−0.4913 to 0.4446	> 0.9999	ns

MMP-1/TIMP-1 ratio, 72 h

R^2 = 0.8935; Tukey's multiple comparisons test; n = 3 independent biological replicates.

High-glucose groups

Comparison	Mean diff.	95% CI	Adjusted P	
HG control vs HG+FA25	0.1500	0.0707 to 0.2293	**0.0002**	***
HG control vs HG+FA50	0.1733	0.0940 to 0.2527	**< 0.0001**	****
HG control vs Mannitol	0.1733	0.0940 to 0.2527	**< 0.0001**	****
HG+FA25 vs HG+FA50	0.0233	−0.0560 to 0.1027	0.9447	ns
HG+FA25 vs Mannitol	0.0233	−0.0560 to 0.1027	0.9447	ns
HG+FA50 vs Mannitol	0.0000	−0.0793 to 0.0793	> 0.9999	ns

Normal-glucose groups

Comparison	Mean diff.	95% CI	Adjusted P	
NG control vs NG+FA25	0.0500	−0.0293 to 0.1293	0.3765	ns
NG control vs NG+FA50	0.0433	−0.0360 to 0.1227	0.5306	ns
NG+FA25 vs NG+FA50	−0.0067	−0.0860 to 0.0727	0.9999	ns
Mannitol vs NG control	−0.0067	−0.0860 to 0.0727	0.9999	ns
Mannitol vs NG+FA25	0.0433	−0.0360 to 0.1227	0.5306	ns
Mannitol vs NG+FA50	0.0367	−0.0427 to 0.1160	0.6971	ns

Controls

Comparison	Mean diff.	95% CI	Adjusted P	
HG control vs NG control	0.1667	0.0873 to 0.2460	**0.0001**	***
HG control vs Mannitol	0.1733	0.0940 to 0.2527	**< 0.0001**	****
Mannitol vs NG control	−0.0067	−0.0860 to 0.0727	0.9999	ns

Remaining Wound Area, 24 h (Normal glucose)

$R^2 = 0.3153$; Tukey's multiple comparisons test; n = 3 independent biological replicates.

Comparison	Mean diff.	95% CI	Adjusted P	
NG control vs 25 FA	−10.24	−33.17 to 12.70	0.5170	ns
NG control vs 50 FA	−2.714	−25.65 to 20.22	0.9802	ns
NG control vs Mannitol	−4.415	−27.35 to 18.52	0.9239	ns
25 FA vs 50 FA	7.525	−15.41 to 30.46	0.7265	ns
25 FA vs Mannitol	5.824	−17.11 to 28.76	0.8467	ns
50 FA vs Mannitol	−1.701	−24.64 to 21.23	0.9949	ns

Remaining Wound Area, 48 h (Normal glucose)

$R^2 = 0.7369$; Tukey's multiple comparisons test; n = 3 independent biological replicates.

Comparison	Mean diff.	95% CI	Adjusted P	
NG control vs 25 FA	−15.38	−35.21 to 4.439	0.1372	ns
NG control vs 50 FA	−21.95	−41.77 to −2.128	**0.0309**	*
NG control vs Mannitol	2.853	−16.97 to 22.68	0.9655	ns
25 FA vs 50 FA	−6.567	−26.39 to 13.26	0.7210	ns
25 FA vs Mannitol	18.24	−1.586 to 38.06	0.0718	ns

Comparison	Mean diff.	95% CI	Adjusted P	
50 FA vs Mannitol	24.80	4.981 to 44.63	**0.0165**	*

Remaining Wound Area, 72 h (Normal glucose)

R^2 = 0.5800; Tukey's multiple comparisons test; n = 3 independent biological replicates.

Comparison	Mean diff.	95% CI	Adjusted P	
NG control vs 25 FA	−6.165	−27.61 to 15.27	0.7949	ns
NG control vs 50 FA	−16.63	−38.07 to 4.806	0.1373	ns
NG control vs Mannitol	4.300	−17.14 to 25.74	0.9153	ns
25 FA vs 50 FA	−10.47	−31.91 to 10.97	0.4478	ns
25 FA vs Mannitol	10.47	−10.98 to 31.91	0.4481	ns
50 FA vs Mannitol	20.93	−0.506 to 42.37	0.0556	ns

Remaining Wound Area, 24 h (High Glucose)

R^2 = 0.3518; Tukey's multiple comparisons test; n = 3 independent biological replicates.

Comparison	Mean diff.	95% CI	Adjusted P	
HG control vs 25 FA	−11.28	−36.34 to 13.78	0.5105	ns
HG control vs 50 FA	−2.962	−28.02 to 22.10	0.9802	ns
HG control vs Mannitol	−9.067	−34.12 to 15.99	0.6668	ns
25 FA vs 50 FA	8.321	−16.74 to 33.38	0.7196	ns
25 FA vs Mannitol	2.216	−22.84 to 27.27	0.9915	ns
50 FA vs Mannitol	−6.105	−31.16 to 18.95	0.8614	ns

Remaining Wound Area, 48 h (High Glucose)

$R^2 = 0.6362$; Tukey's multiple comparisons test; n = 3 independent biological replicates.

Comparison	Mean diff.	95% CI	Adjusted P	
HG control vs 25 FA	−9.094	−29.90 to 11.71	0.5330	ns
HG control vs 50 FA	−6.884	−27.69 to 13.92	0.7217	ns
HG control vs Mannitol	12.94	−7.863 to 33.75	0.2668	ns
25 FA vs 50 FA	2.210	−18.59 to 23.01	0.9855	ns
25 FA vs Mannitol	22.04	1.231 to 42.84	0.0583	ns
50 FA vs Mannitol	19.83	−0.979 to 40.63	0.0618	ns

Remaining Wound Area, 72 h (High Glucose)

$R^2 = 0.8722$; Tukey's multiple comparisons test; n = 3 independent biological replicates.

Comparison	Mean diff.	95% CI	Adjusted P	
HG control vs 25 FA	−7.962	−27.37 to 11.44	0.5800	ns
HG control vs 50 FA	−36.05	−55.45 to −16.64	**0.015**	*
HG control vs Mannitol	4.835	−14.57 to 24.24	0.8536	ns
25 FA vs 50 FA	−28.08	−47.49 to −8.679	0.0532	ns
25 FA vs Mannitol	12.80	−6.608 to 32.20	0.2281	ns
50 FA vs Mannitol	40.88	21.48 to 60.29	0.117	ns
